# The burden of breast cancer in Italy: mastectomies and quadrantectomies performed between 2001 and 2008 based on nationwide hospital discharge records

**DOI:** 10.1186/1756-9966-31-96

**Published:** 2012-11-20

**Authors:** Prisco Piscitelli, Maddalena Barba, Massimo Crespi, Massimo Di Maio, Antonio Santoriello, Massiliamo D’Aiuto, Alfredo Fucito, Arturo Losco, Francesca Pentimalli, Pasquale Maranta, Giovanna Chitano, Alberto Argentiero, Cosimo Neglia, Alessandro Distante, Gian luca Di Tanna, Maria Luisa Brandi, Alfredo Mazza, Ignazio R Marino, Antonio Giordano

**Affiliations:** 1 Department of Internal Medicine, University of Florence, Viale Pieraccini 18, Florence, 50139, Italy; 2 Scientific Direction, Regina Elena National Cancer Institute, Via Elio Chianesi 53, 00144 Rome , 00144, Italy; 3 Epidemiology, Regina Elena National Cancer Institute, Via Elio Chianesi 53, 00144 Rome, 00144, Italy; 4 Local Health Authority of Naples (ASL NA1), Centro piazza Nazionale 95, Naples, 80143, Italy; 5 Department of General Surgery, Second University of Naples, Via Sergio Pansini, Naples, 80131, Italy; 6 Department of Senology, National Cancer Institute G. Pascale Foundation, Via Mariano Semmola, Naples, 80131, Italy; 7 Sbarro Institute for Cancer Research and Molecular Medicine and Center of Biotechnology, College of Science and Technology Temple University, BioLife Science, Bldg. Suite 431 1900 N 12th Street, Philadelphia, PA, 19122, USA; 8 Radiotherapy, Local Health Authority Salerno, via Nizza 146, Salerno, 84124, Italy; 9 INT-CROM, National Cancer Institute G. Pascale Foundation - Cancer Research Center, Via Ammiraglio Bianco, Mercogliano, Avellino, 83013, Italy; 10 Human Health Foundation, Piazza Pianciani 5, Spoleto-Perugia, 06049, Italy; 11 Euro Mediterranean Biomedical Institute (ISBEM) Research Centre, Via Reali di Bulgaria, Brindisi, Mesagne, 72023, Italy; 12 Department of Public Health and Infectious Diseases, La Sapienza University of Rome, Piazzale Aldo Moro 5, Rome, 00185, Italy; 13 Department of Surgery, Jefferson Medical College, Thomas Jefferson University, Philadelphia, PA 19107, USA; 14 Department of Human Pathology & Oncology, University of Siena, Strada delle Scotte, Siena, 53100, Italy

**Keywords:** Hospital discharge records, Breast cancer, Mastectomies, Quadrantectomies, Cancer surveillance

## Abstract

**Background:**

Where population coverage is limited, the exclusive use of Cancer Registries might limit ascertainment of incident cancer cases. We explored the potentials of Nationwide hospital discharge records (NHDRs) to capture incident breast cancer cases in Italy.

**Methods:**

We analyzed NHDRs for mastectomies and quadrantectomies performed between 2001 and 2008. The average annual percentage change (AAPC) and related 95% Confidence Interval (CI) in the actual number of mastectomies and quadrantectomies performed during the study period were computed for the full sample and for subgroups defined by age, surgical procedure, macro-area and singular Region. Re-admissions of the same patients were separately presented.

**Results:**

The overall number of mastectomies decreased, with an AAPC of −2.1% (−2.3 -1.8). This result was largely driven by the values observed for women in the 45 to 64 and 65 to 74 age subgroups (−3.0%, -3.4 -3.6 and −3.3%, -3.8 -2.8, respectively). We observed no significant reduction in mastectomies for women in the remaining age groups. Quadrantectomies showed an overall +4.7 AAPC (95%CI:4.5–4.9), with no substantial differences by age. Analyses by geographical area showed a remarkable decrease in mastectomies, with inter-regional discrepancies possibly depending upon variability in mammography screening coverage and adherence. Quadrantectomies significantly increased, with Southern Regions presenting the highest average rates. Data on repeat admissions within a year revealed a total number of 46,610 major breast surgeries between 2001 and 2008, with an overall +3.2% AAPC (95%CI:2.8-3.6).

**Conclusions:**

In Italy, NHDRs might represent a valuable supplemental data source to integrate Cancer Registries in cancer surveillance.

## Background

Cancer incidence data are a cornerstone of epidemiology research, health monitoring and resource allocation for interventions aimed at cancer prevention and control. Cancer Registries (CRs) contribute to cancer surveillance at local level, throughout the process of systematic collection of data about the occurrence and characteristics of reportable neoplasms [1]. In United States, the National cancer statistics are built on data from a network of CRs called the Surveillance, Epidemiology and End Results Program (SEER). The SEER has now expanded its coverage to 26% of the total population of the United States, accounting for 65.4 million people. Registries included in the SEER share requirements in data reporting and verification procedures throughout a quality improvement process restructured in year 2000. However, the exclusive use of CRs poses limits to the nationwide ascertainment of incident cancer cases, with major concerns arising from the percentage of US population still uncovered [2].

Various secondary databases have been proposed as potential tools to enhance the detection of incident cases and related treatments for a number of diseases, including cancer [3-6]. The accuracy of secondary data sources in capturing cases has been explored with results varying upon the source selected and gold standard used [6-9]. In the study from Penberthy et al., the Virginia Cancer Registry (CR) and a statewide hospital discharge file (HDF) were both tested for accuracy in correctly identifying a cancer and its site of origin. Data from inpatient medical records were used as the gold standard. Based on the conclusions stated, nor the CR neither the HDF was sufficient independently to allow the complete capture of incident cancer cases. However, HDF accuracy in capturing incident cancer cases was high, with the overall positive predictive value being 94% and site specific values ranging from 86% (cervix) to 98% (breast) [9]. In Italy, the government supports cancer surveillance throughout a network of population-based local CRs included in the Italian Association of Cancer Registries (AIRTUM). Currently, the AIRTUM covers 33.8% of the Italian population, namely 19 million people out of 61 million inhabitants. A notable disproportion in CRs coverage exists among Northern, Central and Southern areas of Italy (i.e., 50.2%, 25.5% and 17.9%, respectively) [10].

We have previously underlined the need to integrate data from the Italian CRs with additional sources and identified the National Hospital Discharge Records (NHDRs) as a potential tool [11].

In this study we aimed to evaluate the burden of breast cancer in Italian women by analyzing data from the NHDRs through a non-model-based methodology with a specific focus on major surgical procedures. Compared to our previous work, data have been updated to reflect a larger time window (2001–2008 vs. 2000–2005) and methods refined to overcome some of the limitations from our previous study.

## Materials and methods

### Data source

We used the NHDR database which includes records from all the Italian public and private hospitals. Data were made available by the Italian Ministry of Health relatively to the time frame between 2001 and 2008. These data were subject to a systematic quality assessment performed at a Regional and central level. The matching with the National Institute for Statistics (ISTAT) by social security code showed a percentage of correct linkage increasing from 95.6% in 2001 (50,921 records matched out of 53,226) to 99.8% in 2008 (58,367 records matched out of 58,492) [12,13]. The years 1999 and 2000 were excluded due to incomplete data.

Breast cancer cases were identified on the basis of the International Classification of Diseases, Ninth Revision, Clinical Modification (ICD-9-CM) [14,15]. We considered patients diagnosed with invasive breast cancer (i.e., malignant neoplasm of breast, ICD-9CM codes: 174.0-174.9 and 175.0-175.9). Data related to patients with in situ breast carcinoma (ICD-9-CM major diagnosis 233) were also included.

### Population

Eligible women were patients diagnosed with incident, histologically-confirmed breast cancer who underwent major breast surgical procedures between 2001 and 2008, as identified based on the following codes from the ICD-9-CM: 85.41-48 (mastectomies), 85.22 (quadrantectomies), 85.23 (subtotal mastectomies) [14,15]. In data analysis, mastectomies and subtotal mastectomies (ICD-9-CM codes: 85.41-48 and 85.23, respectively) were ascribed to the same category of major breast surgery (i.e., mastectomies). Excision biopsies and tumorectomies (ICD9-CM code 85.21) were not included. Thus, patients with benign lesions were not considered in our analysis. In order to minimize the overlap between prevalent and incident cases, repeated admissions in any calendar year and across different years for the entire time window considered were discounted and reported separately. We included records pertinent to ordinary hospitalization as well as day hospital regimens.

### Statistical analyses

Data were analyzed using STATA/SE version 11 for Windows (StataCorp LP, College Station, TX, USA) and Microsoft Office Excel 2007 (Microsoft Corp, Seattle, WA, USA). The average annual percentage change (AAPC) and related 95% Confidence Interval (CI) in the actual number of mastectomies and quadrantectomies performed during the study period were computed using a Poisson regression model. To describe time trends, we carried out joinpoint regression analysis.

Analyses were performed for the full sample as well as for subgroups defined by type of surgical procedure (mastectomies and quadrantectomies), age (25–39, 40–44, 45–64, 65–74 and ≥75 years old), and geographical area [i.e., Region and macro-areas (Northern, Central and Southern Italy)]. Results by geographical area were presented in a frame including the indicators of extension and adherence to the national breast cancer screening programs [16].

## Results

Mastectomies and quadrantectomies performed in Italy between 2001 and 2008 are reported in Table 1 and Table 2, respectively. The overall number of mastectomies decreased from 15,754 (year 2001) to 14,197 (year 2008), with an AAPC of −2.1% (−2.3 -1.8). This result is largely driven by the values observed for women in the 45 to 64 and 65 to 74 age subgroups (−3.0%, -3.4 -3.6 and −3.3%, -3.8 -2.8, respectively) and, at a lesser extent, in women aged 75 years and older (−1.2%, -1.7 -0.7). We observed no significant reduction in mastectomies for women aged 25–39 years (+0.3%; -0.8–1.3) and 40-44 years (+1.5%; 0.5–2.5).

**Table 1:** **Mastectomies**^**1**^**performed in Italy between 2001 and 2008**

**Age-group**	**2001**	**2002**	**2003**	**2004**	**2005**	**2006**	**2007**	**2008**	***Subtotals***	**AAPC (95%CI)**^**2**^
**25 - 39**	854	819	849	851	800	786	812	921	**6,692**	
										***+0.3 (−0.8; 1.3)***
**40 - 44**	907	875	962	957	927	1008	955	999	***7,590***	
										***+ 1.5 (0.5; 2.5)***
**45 - 64**	5849	5805	5353	5251	4950	4811	4783	4974	***41,776***	
										***−3.0 (−3.4; -3.6)***
**65 - 74**	3870	3802	3646	3596	3310	3193	3129	3178	***27,724***	
										***−3.3 (−3.8; -2.8)***
**75 - 100**	4274	4464	4516	4265	4126	4157	4053	4125	***33,980***	
										***−1.2 (−1.7; -0.7)***
***Subtotals***	***15,754***	***15,765***	***15,326***	***14,920***	***14,113***	***13,955***	***13,732***	***14,197***	***117,762***	
										***−2.1 (−2.3; -1.8)***

Data are reported by age.

^1^Reported data are absolute numbers unless otherwise specified.

^**2**^AAPC (95%CI): Average Annual Percentage Change and 95% Confidence Interval.

**Table 2:** **Quadrantectomies**^**1**^**performed in Italy between 2001 and 2008**

**Age group**	**2001**	**2002**	**2003**	**2004**	**2005**	**2006**	**2007**	**2008**	***Subtotals***	**AAPC (95%CI)**^**2**^
**25 - 39**	1337	1375	1474	1691	1722	1730	1706	1650	***12,685***	
										+3.6 (2.8; 4.3)
**40 - 44**	1664	1839	1886	2216	2296	2473	2510	2610	***17,494***	
										+6.7 (6.0; 7.4)
**45 - 64**	11573	12032	12334	12952	13294	13614	13908	14820	***104,527***	
										+3.4 (3.1; 3.6)
**65 - 74**	5021	5331	5510	5913	6048	6550	6732	7154	***48,259***	
										+5.1 (4.7; 5.5)
**75 - 100**	2545	2912	3139	3336	3624	3936	4103	4566	***28,161***	
										+ 8.1 (7.5; 8.6)
***Subtotals***	***22,140***	***23,489***	***24,343***	***26,108***	***26,984***	***28,303***	***28,959***	***30,800***	***211,126***	
										**+4.7 (4.5; 4.9)**

^**1**^Reported data are absolute numbers unless otherwise specified.

^**2**^AAPC: Average Annual Percentage Change and 95% Confidence Interval.

Data are reported by age groups.

**Figure 1:**
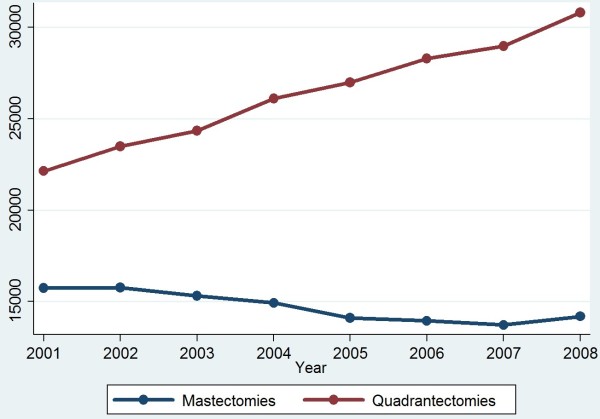
**Temporal Trends in Mastectomies and Quadrantectomies performed in Italy between 2001 and 2008.** Joinpoint analysis for mastectomies and quadrantectomies (absolute numbers) performed in Italy between 2001–8.

As shown in Table 2, there was a +4.7% increase in quadrantectomies (95%CI 4.5-4.9) with the actual numbers rising from 22,140 (year 2001) to 30,800 (year 2008). Temporal trends of mastectomies and quadrantectomies between 2001 and 2008 are shown in Figure 1. Mastectomies were always performed during ordinary hospitalizations, while quadrantectomies performed in a day hospital regimen progressively increased over the 8-year period (+74.2%), accounting for more than 17.5% of the overall breast surgery procedures in 2008 (data available upon request).

In Table 3, we present data by singular Italian Region and macro-areas (i.e., Northern, Central and Southern Italy). Remarkable decreases in the number of mastectomies performed in Italy between 2001 and 2008 were observed in Northern and Central Italy (−2.7%, -3.0 -2.4 and −2.9%, -3.4 -2.4, respectively) but not in Southern Italy (0.3%, -0.3–0.8), where statistically significant reductions were reported for Campania, Calabria and Sicily only.

**Table 3:** **Mastectomies**^**1**^**(Ms) and Quadrantectomies**^**1**^**(Qs) performed in Italy between 2001 and 2008**

**Region**	Mammographic screening coverage (%)*	**Adherence to mammographic screening (%)**^**§**^	**2001**	**2002**	**2003**	**2004**	**2005**	**2006**	**2007**	**2008**	**AAPC (95%CI)**^**2**^
Piemonte Ms	68.6%	65.6%	1222	1177	1138	1146	1112	1140	1053	1032	−2.1 (−2.9; -1.2)
*Qs*			*1686*	*1636*	*1714*	*1856*	*1881*	*2024*	*2160*	*2268*	*+4.9 (4.2; 5.6)*
Valle d'Aosta Ms	92,3%	79,0%	35	26	26	28	16	30	24	23	−4.2 (−9.8; +1.6)
*Qs*			*50*	*62*	*64*	*73*	*76*	*77*	*64*	*72*	*+3.7 (0.0; 7.6)*
Lombardia Ms	92,8%	64,5%	3690	3511	3295	3199	2985	2844	2845	3063	−3.4 (−3.9; -2.9)
*Qs*			*6257*	*6542*	*6428*	*6667*	*6915*	*7048*	*7245*	*7322*	*+2.3 (1.9; 2.7)*
P. A. di Bolzano Ms	n.a.	52,5%	122	113	107	110	93	94	95	89	−4.3 (−7.1; -1.4)
*Qs*			*97*	*69*	*70*	*87*	*78*	*142*	*144*	*175*	*+13.5 (10.2; 17.0)*
P. A. di Trento Ms	80,9%	79,2%	115	127	129	128	146	135	119	134	+1.2 (−1.5; +3.9)
*Qs*			*136*	*175*	*166*	*216*	*208*	*236*	*209*	*251*	*+9.4 (7.5; 11.4)*
Veneto Ms	76,8%	77,1%	1512	1475	1457	1267	1200	1312	1305	1406	−1.8 (−2.6; -1.0)
*Qs*			*1510*	*1612*	*1588*	*1674*	*1595*	*1893*	*2075*	*2296*	*+14.7 (13.8; 15.6)*
Friuli Venezia Giulia Ms	98,7%	62,6%	539	550	571	529	529	534	545	527	−0.5 (−1.8; 0.8)
*Qs*			*533*	*526*	*563*	*606*	*710*	*930*	*809*	*798*	*+8.2 (6.9; 9.4)*
Liguria Ms	34,4%	66,9%	405	393	402	376	420	350	301	334	−3.4 (−4.9; -1.8)
*Qs*			*809*	*847*	*893*	*1.010*	*993*	*1.063*	*1049*	*1077*	*+6.2 (5.1; 7.3)*
Emilia Romagna Ms	96,0%	72,4%	1530	1542	1382	1372	1200	1253	1274	1262	−3.3 (−4.1; -2.5)
*Qs*			*2061*	*2169*	*2148*	*2.378*	*2644*	*2690*	*2666*	*2927*	*+5.2 (4.6; 5.8)*
***Total Northern Italy*Ms**	**82,0%**	**67,9%**	**9,170**	**8,914**	**8,507**	**8,155**	**7,701**	**7,692**	**7,561**	**7,870**	**−2.7 (−3.0; -2.4)**
***Qs***			***13,139***	***13,638***	***13,634***	***14,567***	***15,100***	***16,103***	***16,421***	***17,186***	***+3.3 (3.0; 3.5)***
Toscana Ms	86,4%	69,5%	968	994	841	853	796	814	845	782	−3.0 (−4.0; 2.0)
*Qs*			*1661*	*1859*	*1871*	*2055*	*1960*	*2037*	*2010*	*2022*	*+2.3 (1.6; 3.0)*
Umbria Ms	89,0%	73,3%	249	197	195	216	190	179	161	209	−3.1 (−5.1; -1.0)
*Qs*			*443*	*429*	*453*	*436*	*471*	*501*	*482*	*550*	*+3.1 (1.6; 4.5)*
Marche Ms	74,2%	54,2%	485	515	483	486	472	413	371	378	−4.4 (−5.7; -3.0)
*Qs*			*482*	*537*	*536*	*587*	*653*	*678*	*731*	*753*	*+6.7 (5.4; 8.0)*
Lazio Ms	63,6%	47,6%	*1516*	*1652*	*1456*	*1489*	*1405*	*1382*	*1325*	*1368*	*−2.4 (−3.2; -1.6)*
*Qs*			*2.222*	*2376*	*2581*	*2771*	*2950*	*2759*	*2849*	*3330*	*+4.9 (4.2; 5.5)*
Abruzzo Ms	56,6%	50,5%	267	270	206	225	219	187	217	236	−2.8 (−4.7; -0.8)
			*381*	*375*	*310*	*376*	*332*	*386*	*424*	*421*	*+2.3 (0.7; 3.9)*
***Total Central Italy*Ms**	**78,5%**	**59,7%**	**3,485**	**3,628**	**3,181**	**3,269**	**3,082**	**2,975**	**2,919**	**2,973**	**−2.9 (−3.4; -2.4)**
***Qs***			***5,189***	***5,576***	***5,751***	***6,225***	***6,366***	***6,361***	***6,496***	***7,076***	***+3.9 (3.5; 4.3)***
Molise Ms	48,5%	43,4%	62	55	83	74	69	63	76	47	−1.2 (−4.8; +2.6)
*Qs*			*46*	*70*	*83*	*117*	*103*	*115*	*95*	*121*	*+9.8 (6.4; 13.4)*
Campania Ms	50,0%	29,6%	897	909	950	968	878	786	813	797	−2.4 (−3.4; -1.4)
*Qs*			*1.194*	*1271*	*1323*	*1429*	*1495*	*1568*	*1687*	*1885*	*+6.4 (5.6; 7.3)*
Puglia Ms	25,3%	33,4%	987	928	903	933	901	963	959	1051	+0.9 (0.0; 1.9)
*Qs*			*1.010*	*1174*	*1182*	*1315*	*1324*	*1361*	*1410*	*1520*	*+12.8 (11.7; 13.8)*
Basilicata Ms	100,0%	49,2%	88	98	78	75	89	110	107	114	+4.3 (1.1; 7.6)
*Qs*			*81*	*59*	*92*	*97*	*99*	*110*	*112*	*135*	*+8.9 (5.6; 12.3)*
Calabria Ms	51,8%	26,2%	295	322	320	287	237	239	245	221	−5.1 (−6.9; -3.4)
*Qs*			*195*	*225*	*233*	*302*	*355*	*380*	*362*	*434*	*+11.7 (9.8; 13.7)*
Sicilia Ms	49,2%	41,7%	770	911	856	743	724	719	654	696	−3.4 (−4.5; -2.4)
*Qs*			1.286	1476	1616	1542	1691	1819	1765	1846	+4.6 (3.8; 5.4)
Sardegna Ms	57,2%	54,1%	-	-	448	416	432	408	398	428	−1.1 (−3.4; +1.1)
*Qs*			-	-	429	514	451	486	611	597	+6.7 (4.5; 8.9)
**Total Southern Italy Ms**	**46,5%**	**36,3%**	**3,099**	**3,223**	**3,638**	**3,496**	**3,330**	**3,288**	**3,252**	**3,354**	+**0.3 (−0.3; +0.8)**
***Qs***			***3,812***	***4,275***	***4,958***	***5,316***	***5,518***	***5,839***	***6,042***	***6,538***	***+7.2 (6.8; 7.7)***
**Subtotal ITALY Ms**	***72,7%***	***60,0%***	**15,754**	**15,765**	**15,326**	**14,920**	**14,113**	**13,955**	**13,732**	**14,197**	**−2.1 (−2.3; -1.8)**
***Qs***			***22,140***	***23,489***	***24,343***	***26,108***	***26,984***	***28,303***	***28,959***	***30,800***	***+12.9 (12.7; 13.2)***
**Total ITALY Ms + Qs**			**37,894**	**39,254**	**39,669**	**41,028**	**41,097**	**42,258**	**42,691**	**44,997**	**+2.2 (2.0; 2.3)**

Data are reported by region and macro-area (Northern, Central, and Southern Italy).

^**1**^Reported data are absolute numbers unless otherwise specified.

^**2**^AAPC: Average Annual Percentage Change and 95% Confidence Interval.

*Percentage of women aged 50–69 years old (on total screening target population) invited to perform mammographic screening in 2007–2008 (2-year cumulative data).^18^**§** Adherence rate to mammography screening in year 2008 (adjusted by excluding women performing mammography outside official programs).^16^

Percentages of coverage and adherence to mammographic screening in 2007–08 are also reported.^16^

Quadrantectomies significantly increased across all the Regions but Valle D’Aosta and Abruzzo. When macro-areas were considered, the most remarkable increase was reported for Southern Regions (*+*3.3%, 3.0–3.5;*+*3.9%, 3.5–4.3 and *+*7.2%, 6.8–7.7 for Northern, Central and Southern regions, respectively).

In Table 4, we report mastectomies and quadrantectomies performed on repeated admissions in the same year between 2001 and 2008. Overall, a total number of 46,610 repeated breast surgeries was performed in Italy between 2001 and 2008. Our data showed a significant increase in any of the subcategories considered but the first one (i.e., subcategory including women who underwent repeated breast surgery once within the same year).

**Table 4:** ^**1**^**Mastectomies and**^**1**^**Quadrantectomies performed on repeated admissions between 2001 and 2008**

**Re-interventions (n) in the same patient**	***2001***	***2002***	***2003***	***2004***	***2005***	***2006***	***2007***	***2008***	**AAPC (95%CI)**^**2**^
**1 re-intervention** in the same year	3268	3243	3241	3039	2950	2667	2347	1796	−6.8 (−7.3; -6.3)
**2 re-interventions** in the same year	1387	1981	2419	2834	3092	3484	3560	3794	+12.9 (12.2; 13.5)
**3 re-interventions** in the same year	27	56	132	166	220	240	290	295	+27.5 (24.4; 30.7)
**>3 re-interventions** in the same year	0	0	7	3	17	16	15	24	+45.9 (29.9; 63.9)
**Total Re-interventions**	4682	5280	5799	6042	6279	6407	6212	5909	+3.2 (2.8; 3.6)

Data is presented by categories defined upon the number of repeat major breast surgeries within a year.

^**1**^Reported data are absolute numbers unless otherwise specified.

^2^AAPC: Average Annual Percentage Change (with 95% Confidence Interval, CI).

## Discussion

In the present study, data from the NHDRs proved a valuable tool in the ascertainment of the real figures of incident breast cancer cases. Indeed, the current indications for quadrantectomies or mastectomies in operable breast cancer, along with the use of well defined codes assigned to breast surgeries at the time of patient discharge, render breast cancer particularly prone to traceability through NHDRs. Based on our results, mastectomies decreased in all the age groups but two (i.e., women aged 25–39 and 40–44 years). Conversely, quadrantectomies showed a significant increase across all the age groups. There was a significant decrease in the number of mastectomies in Northern and Central Italy but not in Southern Italy, where the inter-regional differences were remarkable. Quadrantectomies significantly increased across all the different Regions (but Valle D’Aosta and Abruzzo) and macro areas considered.

This study has several strengths. Data were made available by the Italian Ministry of Health. Given that the hospital discharge records provide the basis for hospital care reimbursement within a diagnosis-related groups (DRGs) system, these data are subject to a systematic quality assessment performed at a Regional and central level. Dedicated programs and multidisciplinary workgroups are in place at the Department of Quality Assessment, Management of Medical Care and Ethics of the Italian Ministry of Health to enhance data accuracy and completeness. Constant efforts have led to substantial improvements in data quality. Demographic data accuracy was high. However, inter-regional differences in the completeness of reporting exist and must be taken into account when considering these data [12].

We specifically focused on breast cancer patients having undergone mastectomy or quadrantectomy, whose basic requirement is a histologically-confirmed diagnosis of primary breast cancer. At the same time, we excluded women having undergone excision biopsies and tumorectomies. This approach significantly minimized the inclusion of false positive cases.

Repeated admissions were identified and discounted over the entire 8-year period. This increases our confidence in the ability of the NHDRs to differentiate patients with incident breast cancer cases, included in the present study, from patients with prevalent cancers. Data on repeat admissions were approached in a separate set of analyses (Table 4). Future work will be oriented towards the identification of factors associated with surgery-related hospital readmissions in breast cancer patients, with a specific focus on tumor size and histology, lymph node involvement, type of surgical treatment and patient demographics.

In our analysis, we included data on in situ breast carcinoma. The latter accounted for a small average number of major breast surgeries performed on a yearly basis [i.e., 234 mastectomies (range: 227–301), and 1004 quadrantectomies (range: 725–1300) per year]. In situ breast cancer holds the potentials for malignant transformation. The systematic collection, analysis and reporting of data on carcinoma in situ might help identify risk factors and clarify underlying mechanisms of malignant transformation, thus contributing to breast cancer control research and more targeted treatments [17,18].

Our study has also some limitations. Based on pre-defined selection criteria, our study population includes women eligible for quadrantectomies or mastectomies. The latter category encompasses patients diagnosed with early and locally advanced breast cancer, while generally excluding patients with metastatic breast cancer (MBC) at the time of diagnosis. On this basis, our analysis is expected to underestimate the actual number of breast cancer incident cancer cases. Currently, the percentage of breast cancer patients who are metastatic at diagnosis approximates 6%, with a 5-year survival rate of 21% [19].

We analyzed data related to the time frame spanning from 2001 to 2008. Variations in admitting practices and treatment protocols for the disease of interest might have occurred over time and by area. In few cases, this could have caused discrepancies between the hospital discharges and the actual occurrence of the disease considered [20,21].

Notwithstanding the exclusion of incident cases of metastatic breast cancer (by inclusion criteria), the rates obtained from the analysis of the hospital discharge records were higher than those reported by the Italian Ministry of Health in 2006. According to the CRs 2006 report, the number of estimated breast cancer cases for the year 2006 was 37,542 [22]. In the same year, we observed 42,258 cases (i.e., +11%). Several factors might contribute to such a discrepancy. First, in our study the linking process allowed the discharge of repeat hospital admission between 2001 and 2008, but discharge data related to patients who had been admitted for breast cancer in years prior to 2001 might still be present. Indeed, 10–15 percent of patients undergoing breast conservative therapy for operable breast cancer (i.e., breast-conserving surgery and postoperative breast irradiation) will develop a loco-regional recurrence within 10 years [23]. This risk is slightly higher than that of a loco-regional recurrence following mastectomy (5 to 10 percent) [23,24]. However, these rates include both metastases occurring in the ipsilateral preserved breast (i.e., local recurrence) and regional lymph nodes, (i.e., regional recurrence), with only the first representing a potential target for breast surgery. Second, our analysis included data on carcinoma in situ of the breast, which are not routinely collected and analyzed by CRs [17]. Third, the official estimates were based on the use of the Mortality and Incidence Analysis Model method (MIAMOD), a back-calculation approach which obtains cancer-specific morbidity measures by using official mortality data and model-based relative survival from local cancer registry data. As such, the MIAMOD method reflects the limitations stemming from the incomplete coverage and disproportion among macro-areas which characterize the Italian network of CRs [10]. On this basis, underreporting of cases and, consequently, underestimation of the cancer burden cannot be excluded when using the MIAMOD approach.

Significant increases in quadrantectomies were reported in women aged 25 to 39 and 40 to 44 years. Women in these age groups are still formally uncovered by the breast cancer screening programs activated by the Italian Ministry of Health, despite they represent 13.6% of women undergoing total major breast procedures [16].

In general, our figures showed inverse trends for mastectomies and quadrantectomies performed in Italy between 2001 and 2008. The increase observed for quadrantectomies and the decrease concerning mastectomies might be interpreted in light of the progressive expansion of the screening programs, and the better adherence to updated treatment protocols [16]. Indeed, mammographic screen-detected cancers show more favorable prognostic features at diagnosis and need less extensive treatment compared to symptomatic cancers [25]. The heterogeneous distribution of such interventions (i.e., screening programs), particularly in Southern Italy, might account for the differences in trends across macro areas and singular regions.

Several studies have investigated the use of hospital discharge records to enhance cancer surveillance. In 1996, Huff and co-authors estimated disease occurrence rates from hospital discharge data for breast, cervical and lung cancer at a state- and county level for the state of Maine, US. Consistently with our results, rates from hospital discharge data were higher than rates from cancer registry data. It is noteworthy that the breast cancer rates from NHDRs and Cancer Registry data were the ones with the higher correlation among those considered (correlation coefficients were 0.87, 0.79 and 0.55 for breast, lung and cervical cancer, respectively) [26]. We have previously proposed the use of the NHDRs to evaluate the breast cancer burden in Italy [11]. Results across our two studies are fairly consistent. However, results from our previous study were limited by the inclusion of repeat hospital admissions. Moreover, a different and more restricted time window was considered (i.e., 2000–2005). Ferretti et al. used an algorithm based on Regional hospital discharge records to estimate breast cancer incidence in three Italian regions covered by the Italian net of CRs (e.g., Emilia Romagna, Toscana and Veneto). Incidence rates of the two methods showed no statistical differences. However, the authors ascribed the agreement between hospital discharge records and CRs incidence rates to a cross effect of both sensitivity and specificity limitations of the discharge records algorithm [27].

## Conclusions

A National system of population-based CRs is essential to monitor cancer patterns and trends at a National and local level and to orient health monitoring and resource allocation decisions [28]. However, the exclusive use of CRs may pose limits to the estimate of cancer burden, mainly due to incomplete and heterogeneous coverage. We suggest the use of the NHDRs to supplement the net of CRs. The latter source (NHDRs) may be a valuable and relatively efficient tool for enhancing cancer surveillance.

## Abbreviations

et al: And co-authors; AAPC: Average annual percentage change; CR: Cancer registry; CRs: Cancer registries; CI: Confidence interval; e.g: Exampli gratia; HDF: Hospital discharge file; i.e: Id est; ICD-9-CM: International classification of diseases, ninth revision, clinical modification; AIRTUM: Italian association of cancer registries; Ms: Mastectomies; MIAMOD: Mortality and incidence analysis model; NHDRs: National hospital discharge Records; ISTAT: National Institute for Statistics; Qs: Quadrantectomies; SEER: Surveillance, epidemiology and end results program; US: United states.

## Competing interests

The authors declare that they have no competing interests.

## Authors’ contributions

PP, study conception and design, data acquisition, manuscript drafting. MB, manuscript drafting, methodological advise. MC, critical revision for important intellectual contents. MDM, critical revisions for important intellectual contents. AS, critical revisions for important intellectual contents. MD, critical revisions for important intellectual contents. AF, critical revisions for important intellectual contents. AL, critical revisions for important intellectual contents. FP, critical revisions for important intellectual contents. PM, critical revisions for important intellectual contents. GC, critical revisions for important intellectual contents; AA, critical revisions for important intellectual contents. CN, critical revisions for important intellectual contents. AD, critical revisions for important intellectual contents. GDT, data analysis, results’ interpretation. MLB, critical revisions for important intellectual contents. AM, critical revisions for important intellectual contents. IRM, data acquisition, methodological advise. AG, study conception and design, methodological advice. All authors read and approved the final manuscript.
